# Resveratrol Attenuates Neurodegeneration and Improves Neurological Outcomes after Intracerebral Hemorrhage in Mice

**DOI:** 10.3389/fncel.2017.00228

**Published:** 2017-08-08

**Authors:** Frederick Bonsack, Cargill H. Alleyne, Sangeetha Sukumari-Ramesh

**Affiliations:** Department of Neurosurgery, Medical College of Georgia, Augusta University Augusta, GA, United States

**Keywords:** neurological outcomes, cerebral edema, ICH, hematoma, resveratrol

## Abstract

Intracerebral hemorrhage (ICH) is a devastating type of stroke with a substantial public health impact. Currently, there is no effective treatment for ICH. The purpose of the study was to evaluate whether the post-injury administration of Resveratrol confers neuroprotection in a pre-clinical model of ICH. To this end, ICH was induced in adult male CD1 mice by collagenase injection method. Resveratrol (10 mg/kg) or vehicle was administered at 30 min post-induction of ICH and the neurobehavioral outcome, neurodegeneration, cerebral edema, hematoma resolution and neuroinflammation were assessed. The Resveratrol treatment significantly attenuated acute neurological deficits, neurodegeneration and cerebral edema after ICH in comparison to vehicle treated controls. Further, Resveratrol treated mice exhibited improved hematoma resolution with a concomitant reduction in the expression of proinflammatory cytokine, IL-1β after ICH. Altogether, the data suggest the efficacy of post-injury administration of Resveratrol in improving acute neurological function after ICH.

## Introduction

Intracerebral hemorrhage (ICH) is a catastrophic type of stroke caused by bleeding within the brain parenchyma (Leclerc et al., 2015). Approximately, 10–15% of strokes are caused by ICH. Despite recent advances in clinical and preclinical research, the one-month mortality rate of ICH is 40% and only about 20% of the survivors with spontaneous ICH regain functional independence at 6 months (Flemming et al., 2001; Qureshi et al., 2001; Gebel et al., 2002; Flaherty et al., 2006; Ke et al., 2015). Primary as well as secondary brain damage is involved in the pathological processes of ICH. The primary damage usually occurs within minutes to hours and is mainly caused by mechanical disruption resulting from the mass effect of hematoma, whereas the cytotoxicity of blood, excitotoxicity, oxidative stress, and inflammation together result in secondary brain damage, causing severe disability or death (Xi et al., 2006; Aronowski and Zhao, 2011). Notably, there is no effective therapeutic or surgical treatment for ICH and the current treatment options even in dedicated stroke centers are limited to supportive care. Therefore, finding new treatment regimens that could provide safety and neuroprotection to patients suffering from ICH is critical.

Resveratrol (3,5,4′-trihydroxystilbene) is a naturally occurring polyphenolic compound and the content of Resveratrol in major dietary sources such as grapes and red wine ranges from 0.16 to 3.54 μg/g and 0.1 to 14.3 mg/L, respectively (Mark et al., 2005; Baur and Sinclair, 2006; Mukherjee et al., 2010). Resveratrol is associated with anti-inflammatory, anti-oxidant, anti-apoptotic properties (Narayanan et al., 2015; Taguchi et al., 2015; Tellone et al., 2015; Tsai et al., 2015; Gwak et al., 2016; Hoda et al., 2016) and is able to cross the blood–brain barrier (BBB) making it an ideal candidate to be tested for its role in neuropathological conditions. In addition, Resveratrol has been tested for the treatment of various neuroinflammatory and neurodegenerative diseases such as stroke, spinal cord injury, epilepsy, Huntington's disease and Alzheimer's disease (Gupta et al., 2001, 2002; Wang et al., 2002; Yang and Piao, 2003; Kiziltepe et al., 2004; Kaplan et al., 2005; Parker et al., 2005; Ates et al., 2006; West et al., 2007; Wu et al., 2009; Li et al., 2014; Shao et al., 2014; Lopez et al., 2015), and it was well tolerated in preclinical animal models. However, the neuroprotective efficacy of Resveratrol after ICH remains largely unstudied. Further, to date, very few studies have reported whether post-injury administration of resveratrol can protect against brain injury. Therefore, the main objective of the present study is to evaluate whether the post-treatment with Resveratrol confers neuroprotection in a pre-clinical model of ICH.

## Materials and methods

### ICH

Animal studies were reviewed and approved by the Committee on Animal Use for Research and Education at Augusta University, in compliance with NIH and USDA guidelines. ICH was induced in adult male CD-1 mice (Charles River) as previously reported (Sukumari-Ramesh et al., 2012a,b, 2016; Bonsack et al., 2016; Sukumari-Ramesh and Alleyne, 2016). Briefly, mice (*n* = 72) were anesthetized with ketamine and xylazine and prone-positioned on a stereotaxic head frame (Stoelting, WI, U.S.A.). The body temperature was maintained at 37 ± 0.5°C during the surgical procedure using a small animal temperature controller (David Kopf Instruments, USA) and a burr hole (0.5 mm) was made 2.2 mm lateral to bregma using a high-speed drill (Dremel, USA) without damaging the underlying dura. A Hamilton syringe (26-G) containing 0.04U of bacterial type IV collagenase (Sigma, St. Louis, MO) in 0.5 μL phosphate buffered saline (pH 7.4; PBS) was inserted with stereotaxic guidance 3.0 mm into the left striatum to induce spontaneous ICH (Bonsack et al., 2016). After removal of the needle, the burr hole was sealed with bone wax and the incision was stapled. Sham mice underwent the same surgical procedure, but only PBS (0.5 μL) was injected.

### Administration of resveratrol

Resveratrol was purchased from Sigma (St. Louis, MO, USA). Resveratrol (10 mg/kg), freshly prepared in a 1:2 solution of DMSO: PBS, was administered intravenously (tail vein) in a total volume of 100 μl at 30 min post-induction of ICH and the control mice received equal volume of vehicle (DMSO) in PBS.

### Immunohistochemistry

After being anesthetized, mice were transcardially perfused with PBS. Brains were collected, fixed with 4% paraformaldehyde, snap frozen, and cut into coronal sections (25 μM) using a cryostat. Sections (*n* = 3–4/group) were then mounted onto glass slides and incubated with 10% normal donkey serum for 2 h at room temperature. This was followed by incubation with primary antibody at 4°C for 24 h and subsequent washing as well as incubation with corresponding Alexa Fluor-tagged secondary antibody for 1 h at room temperature. The immunofluorescence was acquired using Zeiss LSM510 Meta confocal laser microscope and 3–6 non-consecutive sections per animal were subjected to analysis.

### Fluoro-jade B staining

Hydrated brain sections (*n* = 3–4/group) were placed in a 0.06% potassium permanganate solution for 15 min and subsequently incubated with 0.001% Fluoro-Jade B solution for 30 min. Sections were allowed to air dry and cover-slipped with DPX mounting media. Microscopic analysis was performed using an excitation wavelength of 488 nm, provided by an argon laser and the images were taken using a LSM510 Meta confocal laser microscope.

### Tunel staining

Cellular apoptosis was detected using a commercially available apoptosis detection kit (Apoptag; Millipore; S7110). Briefly, brain sections (*n* = 3–4/group) were fixed in ethanol; acetic acid and incubated in an equilibration buffer. Sections were then treated with Terminal deoxynucleotidyl transferase (TdT) enzyme in reaction buffer and subsequently incubated with anti-digoxigenin-fluorescein conjugate solution for 30 min at room temperature. The fluorescence was determined using a LSM510 Meta confocal laser microscope.

### Quantitative estimation of fluoro jade B and tunel positive cells

To estimate the number of Fluoro jade B and TUNEL positive cells, the coronal brain sections of thickness 20-μm were subjected to respective staining, as described earlier. We used three sections (one from the collagenase injection site and other two from 0.25 mm anterior and 0.25 mm posterior to the injection site) per animal (*n* = 3–4/group) and examined different areas around the hematoma, as depicted in schematic figure (Figure **2A**). The positive cells were counted with the aid of image J software and the average number of cells per area is provided.

### Cerebral edema

Mice (*n* = 4–8/group) were anesthetized and decapitated after the induction of sham or ICH. The brains were removed, and a coronal brain slice containing cortex and striatum was collected and immediately weighed on an electronic analytical balance to obtain the wet weight. Brain samples were then dried at 100°C for 24 h to obtain the dry weight. The Brain water content (%) was calculated as [(wet weight − dry weight)/wet weight] × 100.

### Neurological outcome

Neurobehavioral outcome (*n* = 9–13/group) was estimated by an independent researcher blinded to the experimental groups using a composite neurological test, as detailed previously by our laboratory and others (Rosenberg et al., 1990; Clark et al., 1998; King et al., 2011; Sukumari-Ramesh and Alleyne, 2016; Sukumari-Ramesh et al., 2016). This 24-point scale composite test that determines the sensorimotor deficits associated with intrastriatal ICH, is comprised of six neurobehavioral sub-tests (climbing, circling, compulsory circling, whisker response, bilateral grasp, and beam walking; Rosenberg et al., 1990; Clark et al., 1998; King et al., 2011; Sukumari-Ramesh and Alleyne, 2016; Sukumari-Ramesh et al., 2016). Briefly, the climbing ability of the mouse was assessed using a gripping surface kept at 45° angle and the circling behavior was tested on an open bench top. To assess compulsory circling, mouse was placed on its front limbs on a bench and held suspended by its tail and the whisker response was evaluated with a gentle touch to its whisker using a swab. The bilateral grasp assessed the strength to hold onto a steel grip-bar with forepaws and the beam walking was graded by evaluating the ability of a mouse to traverse a narrow beam. Each sub-test was scored from 0 (performs with no impairment) to 4 (severe impairment) and the individual subtest scores are provided as Supplementary Data-Table 1. A composite score was calculated as the sum of the scores on all the six sub-tests, establishing a maximum neurological deficit score of 24.

### Cresyl violet staining

Brain sections (*n* = 3–4/group) were rehydrated with ethanol and stained with 0.5% Cresyl Violet Solution. Sections were then dehydrated, treated with xylene and subjected to imaging.

### Hematoma volume

Mice were euthanized and brain of each mouse (*n* = 3–4/group) was cut into coronal sections with a cryostat. Five to six sections per mouse were subjected to cresyl violet staining as detailed earlier and using Image J software (NIH, USA), the area of the hematoma was quantified. The hematoma volume was then calculated by multiplying the sum of the areas by the interslice distance.

### Hemoglobin assay

Hemoglobin assay was conducted as a measure of hematoma volume, as described previously (Wei et al., 2017). Briefly, anesthetized mice (*n* = 5/group) were subjected to complete transcardial perfusion with PBS to remove intravascular blood and brain tissue was collected. The respective ipsi- and contralateral brain sections were homogenized in PBS, centrifuged at 10,000 g for 15 min at 4°C and the supernatant was subjected to hemoglobin assay using a Hemoglobin Colorimetric Assay Kit (Cayman, Ann Arbor, USA). The amount of heme as a measure of hemoglobin content was calculated using a standard curve generated from known heme values, as per manufacture's instructions.

### Statistical analysis

Mice were randomly assigned to the experimental groups, and all analyses were performed by an investigator-blinded manner. Data were analyzed using unpaired *t*-test or one-way analysis of variance followed by Student-Newman-Keuls *post hoc* test, as appropriate and are expressed as mean ± SE. A *p* value of <0.05 was considered as significant.

## Results

### Resveratrol improved acute neurological outcomes after ICH

**Figure 1:**
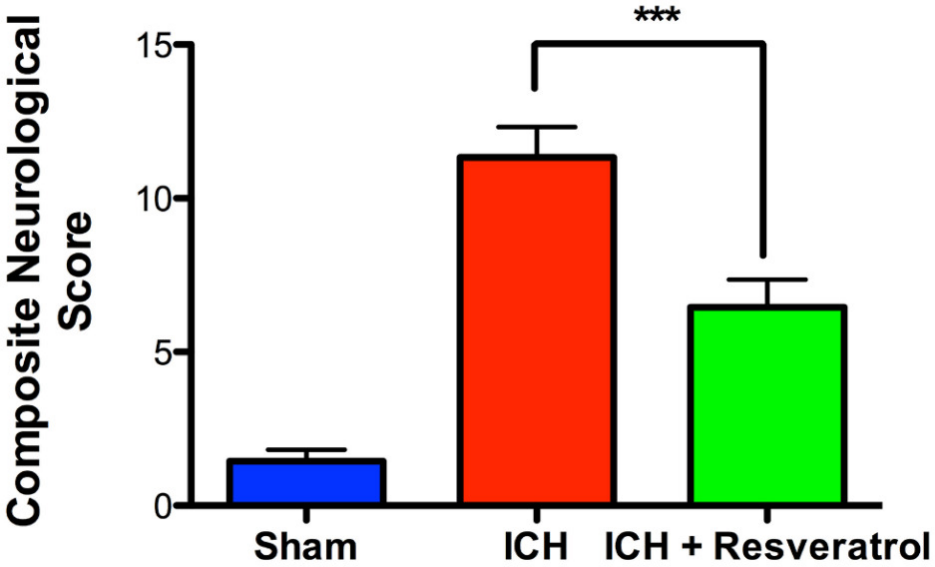
Resveratrol improved acute neurological outcomes at 24 h after ICH. Resveratrol (10 mg/kg; i.v) was administered at 30 min after the induction of ICH and neurobehavioral outcome was estimated at 24 h post injury, employing a composite neurological test and Resveratrol treatment significantly reduced ICH-induced neurobehavioral deficits (^***^p < 0.001). *n* = 9–13/group.

To determine the neuroprotective efficacy of Resveratrol after ICH, Resveratrol 10 mg/kg (i.v) was administered 30 min after the induction of ICH in mice and the control mice received equal volume of vehicle (DMSO) in PBS. ICH-induced mice exhibited significant neurobehavioral deficits 24 h post injury in comparison to sham as estimated using a composite neurobehavioral test on a 24 point scale comprised of six neurobehavioral subtests (Figure 1). The Resveratrol treatment significantly attenuated ICH-induced neurobehavioral deficits in comparison to vehicle treated controls and the composite neurological deficit score after ICH was reduced by 42.5% upon Resveratrol treatment in comparison to vehicle treated controls (*p* < 0.001; *n* = 9–13/group; Figure 1).

### Resveratrol attenuated acute neurodegeneration after ICH

**Figure 2:**
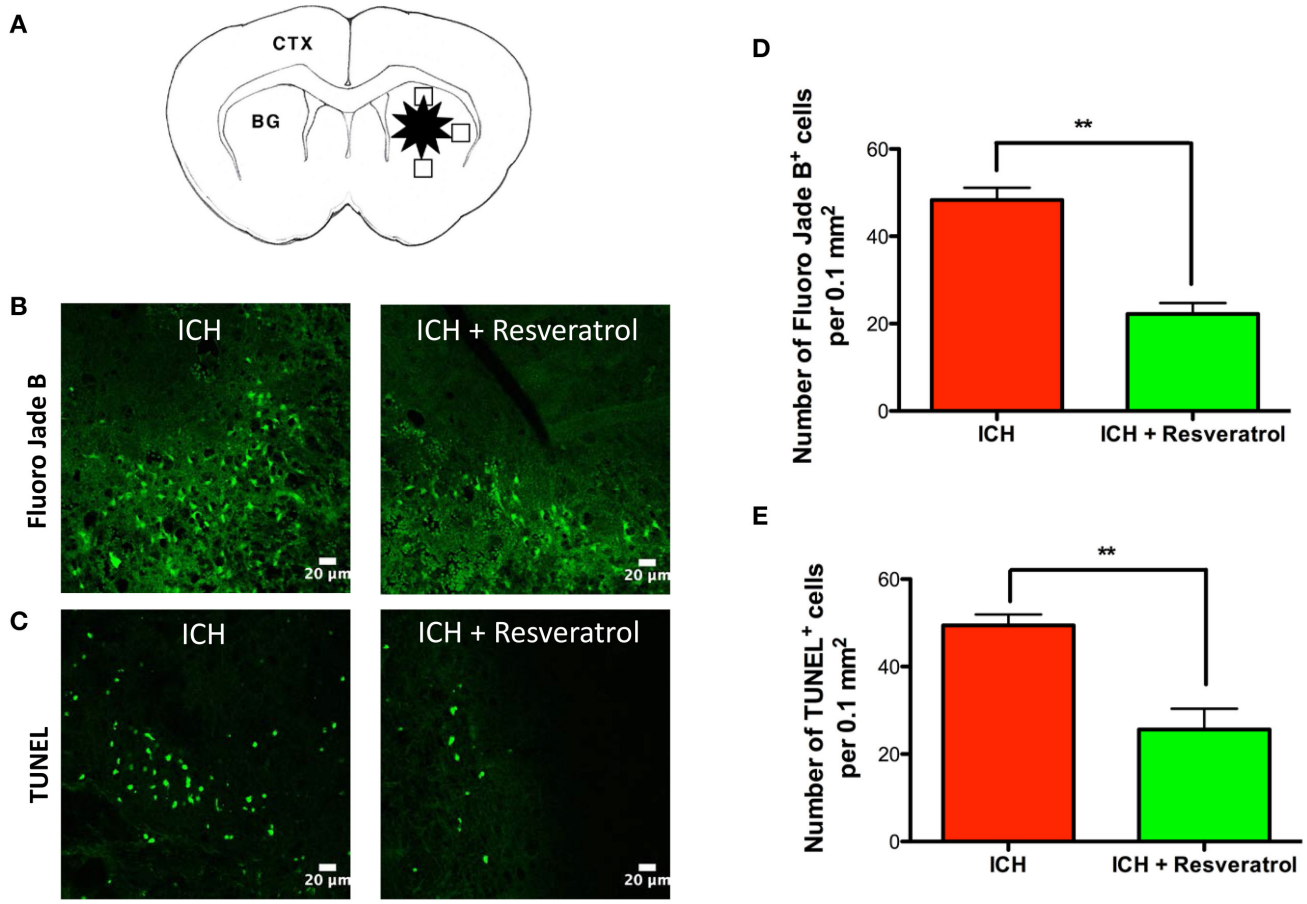
Resveratrol attenuated acute neurodegeneration after ICH. To estimate the number of Fluoro jade B and TUNEL positive cells, we used three coronal sections of thickness 20-μm (one from the collagenase injection site and other two from 0.25 mm anterior and 0.25 mm posterior to the injection site) per animal and the respective staining was performed as described in methods at 24 h post ICH. The schematic diagram **(A)** depicts a coronal brain section and the areas (the square black boxes) around the hematoma that were examined for Fluoro Jade B /TUNEL-positive cells. CTX, cortex; BG, basal ganglia. The positive cells were counted with the aid of image J software and the average number of cells per area is provided. The representative confocal images (scale bar, 20 μm; **B,C**) and the respective quantification demonstrate significantly reduced number of both Fluoro Jade B and TUNEL positive cells after ICH upon Resveratrol (10 mg/kg; i.v) treatment in comparison to vehicle treated controls (^**^p < 0.01; **D,E**). *n* = 3–4/group.

To test whether the Resveratrol-mediated improvement in acute neurological outcome is concomitant with a reduction in neuronal death, the brain sections were subjected to Fluor Jade B staining, which recognizes degenerated neurons. Post-injury administration of Resveratrol significantly attenuated the number of Fluoro Jade B-positive cells after ICH, in comparison to vehicle treated controls suggesting Resveratrol mediated attenuation of acute neurodegeneration after ICH (Figure 2B). This was further confirmed by TUNEL staining (Figure 2C), which recognizes early apoptotic cells including neurons and glial cells. Notably, the number of Fluoro Jade B as well as TUNEL positive cells was reduced by 54 and 48.1% respectively, upon Resveratrol treatment in comparison to vehicle treated controls (*p* < 0.01; *n* = 3–4/group) (Figures 2D,E).

### Resveratrol treatment attenuated cerebral edema after ICH

**Figure 3:**
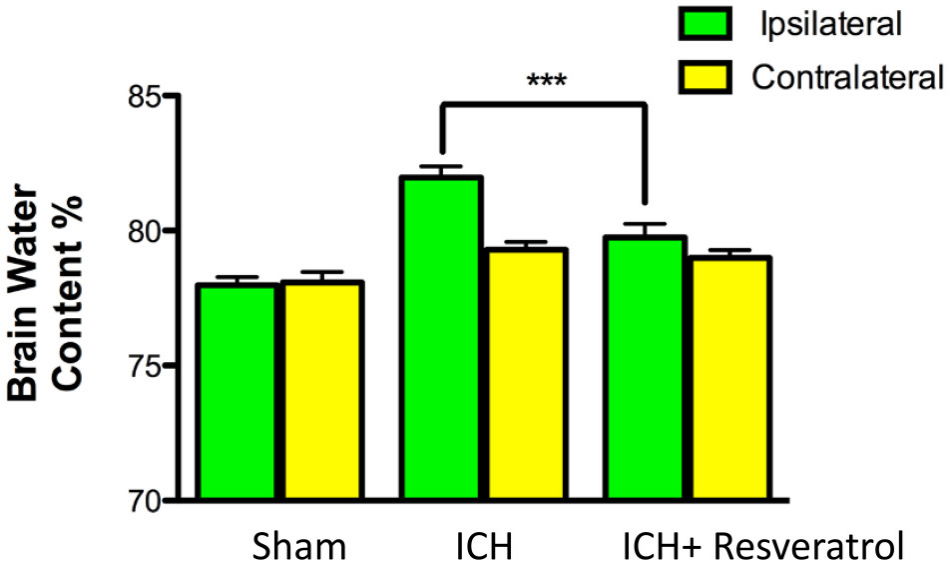
Resveratrol treatment attenuated cerebral edema after ICH. Resveratrol (10 mg/kg; i.v) significantly reduced the brain water content in the ipsi-lateral brain region after ICH in comparison to the vehicle treated ICH as assessed by wet/dry method as described in the methods section (^***^p < 0.001). No significant differences in the brain water content were observed in the contralateral hemisphere between the two groups. *n* = 4–8/group.

Given the detrimental role of cerebral edema in ICH-induced brain damage and neurological outcome (Wang and Tsirka, 2005; Xi et al., 2006; Wasserman and Schlichter, 2007), we next questioned whether Resveratrol treatment could attenuate cerebral edema after ICH. To test this, the brain water content was estimated and there was a significant increase in brain water content in the ipsilateral brain region after ICH in comparison to sham (Figure 3). However, no significant increase in brain water content was observed in the contralateral brain region after ICH (Figure 3). Notably, Resveratrol treatment significantly attenuated ICH-induced cerebral edema in the ipsilateral brain region in comparison to vehicle treated controls (Figure 3). Along these lines, the brain water content in the vehicle-treated ICH group was 81.96 ± 0.42% whereas the Resveratrol-treated group exhibited brain water content of 79.74 ± 0.51% (*p* < 0.001 vs. vehicle treated ICH; *n* = 4–8/group; Figure 3).

### Resveratrol attenuated hematoma volume and IL-1 β expression after ICH

**Figure 4:**
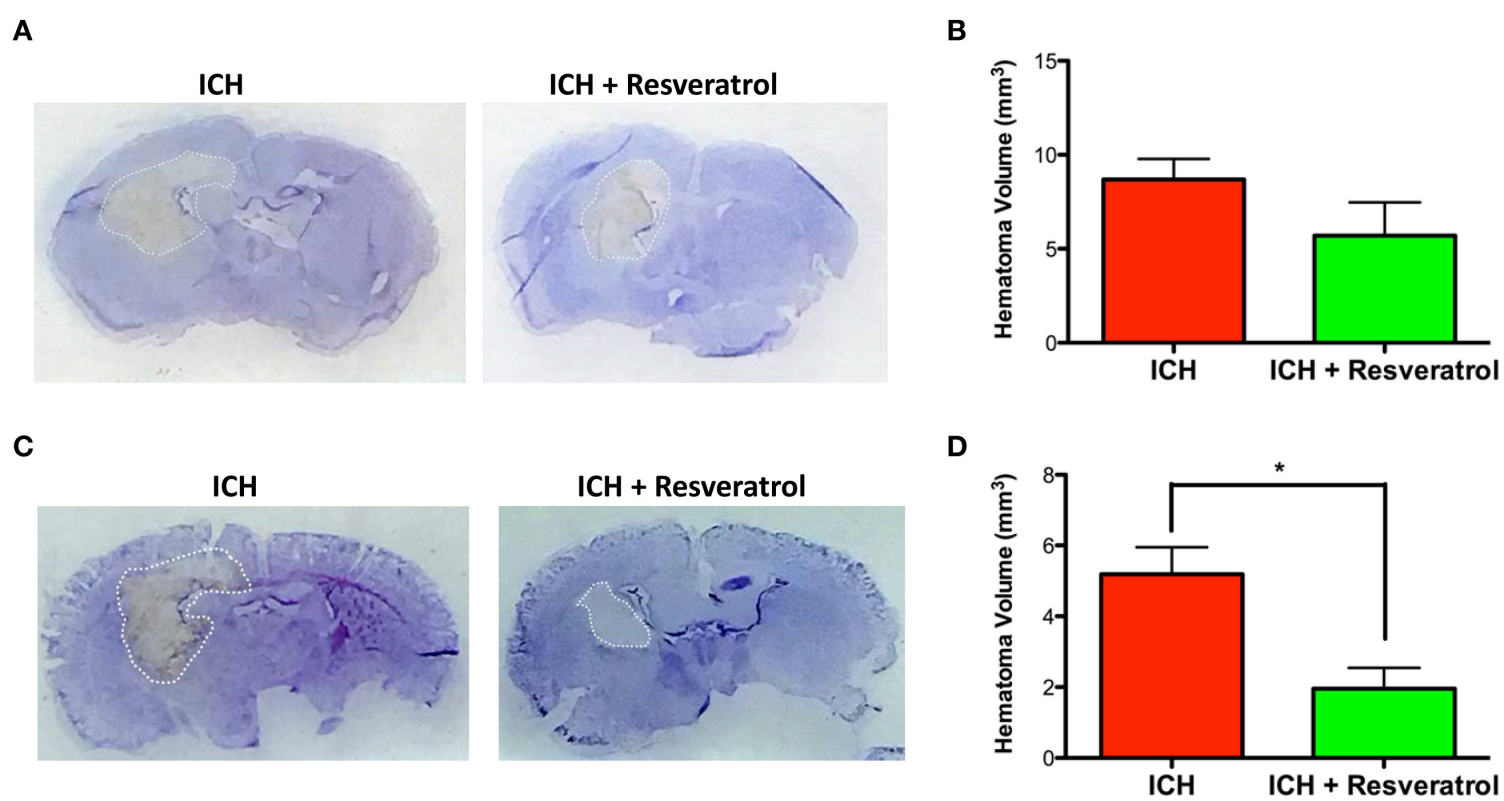
Resveratrol attenuated hematoma volume after ICH. Brain sections were stained with Cresyl Violet at 24 or 72 h post-ICH to illustrate the area of hematoma **(A,C)**. Hematoma volume was calculated as described in the methods and Resveratrol treatment significantly reduced the hematoma volume at 72 h post-ICH in comparison to vehicle treated controls (^*^p < 0.05; **D**) whereas no significant reduction in hematoma volume was observed at 24 h post-ICH **(B)**. *n* = 3–4/group.

**Figure 5:**
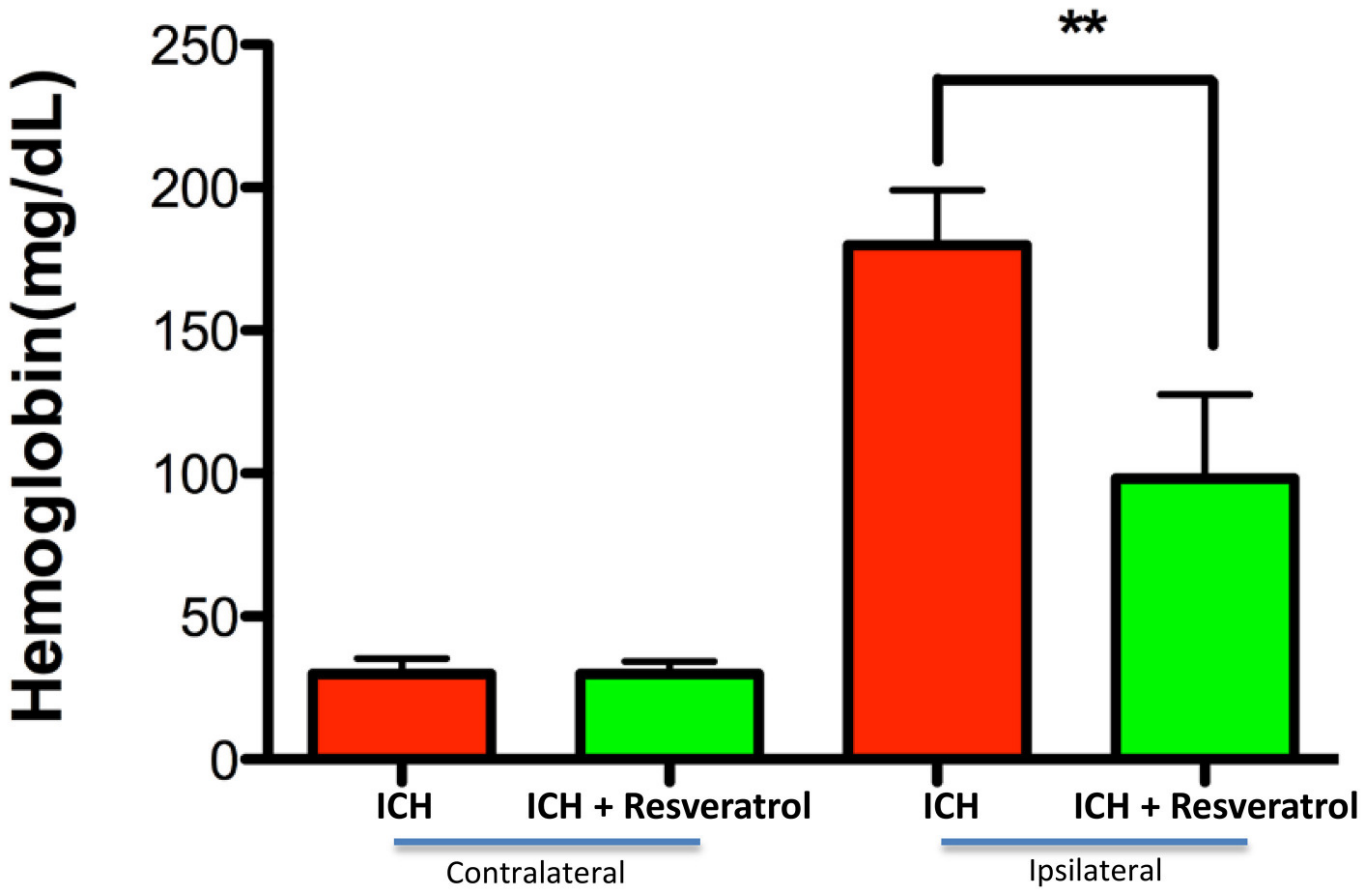
Resveratrol improved hematoma resolution after ICH. To assess hematoma volume, the hemoglobin content in the brain was estimated using a hemoglobin assay kit and there was a significant decrease in hemoglobin content at 72 h post-ICH in the ipsi lateral brain region upon Resveratrol treatment in comparison to vehicle treated controls (^**^p < 0.01) whereas no reduction in hemoglobin content was observed in the contra lateral brain region. *n* = 5/group.

**Figure 6:**
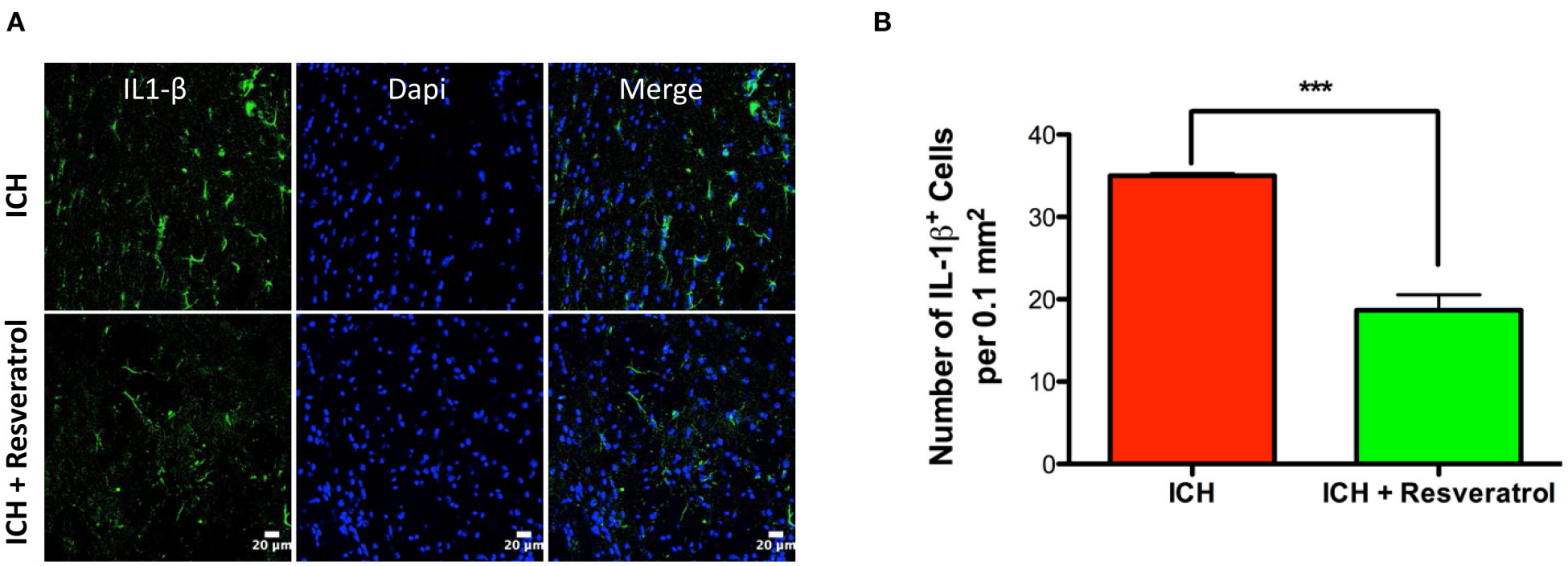
Resveratrol attenuated IL-1 β expression after ICH. Brain sections from Resveratrol or vehicle treated ICH mice were immunostained for IL-1 β and cover slipped with a mounting media containing Dapi, a nuclear stain. The confocal images for IL-1 β staining are demonstrated **(A)**. Resveratrol treatment significantly reduced the number of IL-1 β positive cells in the peri-hematomal brain region in comparison to vehicle treated control (^***^p < 0.001; **B**). *n* = 3–4 /group.

**Figure 7:**
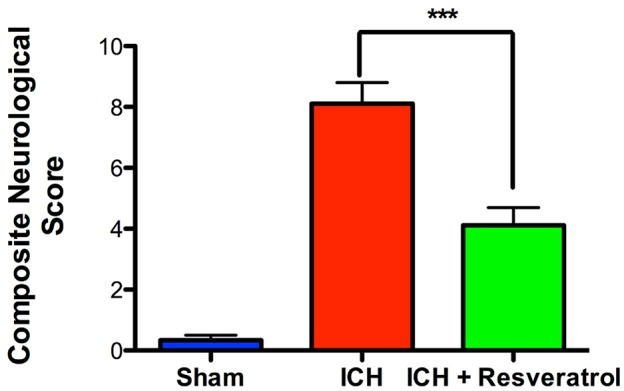
Resveratrol improved acute neurological outcomes at 72 h after ICH. Resveratrol (10 mg/kg; i.v) was administered at 30 min after the induction of ICH and neurobehavioral outcome was estimated at 72 h post injury, employing a composite neurological test and Resveratrol treatment significantly reduced ICH-induced neurobehavioral deficits (^***^p < 0.001). *n* = 9/group.

Hematoma volume is an independent predictor of ICH-induced mortality and neurological deficits (Broderick et al., 1993; Kothari et al., 1996; Flemming et al., 1999; Wang et al., 2014). Of note, Resveratrol treatment significantly reduced the hematoma volume as assessed by Cresyl violet staining at 72 h post injury in comparison to vehicle treated controls (*p* < 0.05; *n* = 3–4/group) further emphasizing the neuroprotective efficacy of Resveratrol after ICH (Figures 4C,D). The reduction in hematoma volume at 72 h post-ICH was further confirmed by hemoglobin assay, that demonstrated a significant reduction in hemoglobin content in the ipsilateral brain region upon Resveratrol treatment in comparison to vehicle treated controls (*p* < 0.01; *n* = 5/group, Figure 5). Notably, no significant reduction in hematoma volume was observed at 24 h post-injury upon Resveratrol treatment (Figures 4A,B) suggesting that Resveratrol could have improved the hematoma resolution rather than modulating the initial hematoma formation after ICH. Further, hematoma components such as erythrocyte lysis products are key modulators of brain inflammation and among the pro-inflammatory cytokines, IL-1β is considered as a pivotal inflammatory target after ICH (Masada et al., 2001; Lok et al., 2012). Therefore, we next questioned whether Resveratrol treatment reduced IL-1β expression at 72 h post ICH, a time point that exhibits profound proinflammatory activation of microglia/macrophages (Sukumari-Ramesh et al., 2012a; Bonsack et al., 2016). There was a significant reduction in IL-1 β positive cells after ICH upon resveratrol treatment in comparison to vehicle treated controls (*p* < 0.001; *n* = 3–4/group, Figures 6A,B). In addition, the reduction in hematoma volume and the number of IL-1 β positive cells upon Resveratrol treatment was concomitant with a significant improvement in neurobehavioral outcome at 72 h post-ICH in comparison to vehicle-treated control (*p* < 0.001; *n* = 9/group, Figure 7).

## Discussion

ICH is a major healthcare concern with highest mortality rates among all types of stroke. Further, if the ICH patients survive they often exhibit poor clinical prognosis demanding effective therapeutic intervention. Herein, we report for the first time that post-injury administration of Resveratrol significantly improved acute neurological outcome, hematoma resolution, and attenuated neurodegeneration in a preclinical model of ICH. Resveratrol-mediated neuroprotection was also associated with a significant reduction in cerebral edema and the expression of key proinflammatory cytokine IL-1β after ICH.

ICH comprises of both primary and secondary brain injury. Currently, the treatment for ICH mainly focuses primarily on supportive care but this clinical approach targeting the primary brain injury often fails to improve neurological outcome or decrease mortality associated with ICH. Notably, secondary brain injury, causing severe neurological deficit or death, proceeds over hours to days after ICH and offers a potential target for therapeutic intervention (Keep et al., 2012; Urday et al., 2015a,b). However, the secondary brain damage after ICH is complex and is caused primarily by the cytotoxic effect of extravasated blood. This cytotoxic insult has a strong oxidative and inflammatory component, ultimately leading to neurological dysfunction and death (Wagner et al., 2003; Aronowski and Hall, 2005; Xi et al., 2006; Hanley, 2009). Therefore, strategies to efficiently remove intraparenchymal blood/erythrocyte lysis components or augment hematoma resolution may attenuate secondary brain damage and reduce the neurotoxicity of hematoma components and associated brain inflammation. To this end, post injury administration of Resveratrol significantly attenuated hematoma volume and the expression of the key proinflammatory cytokine IL-1β after ICH implicating the possible role of Resveratrol in modulating secondary brain damage after ICH.

Brain edema is a common and life-threatening clinical complication after ICH and it causes mass effect on adjacent brain structures, elevation in intracranial pressure, hydrocephalus, brain herniation, and severe neurological deficits (Zheng et al., 2016). The development of brain edema after ICH appears to be partly related to erythrocyte lysis products inducing both oxidative and inflammatory signaling (Hu et al., 2016) and the intracerebral accumulation of serum resulting from clot retraction (Butcher et al., 2004). Accumulating evidence suggest that pharmacological agents that can reduce cerebral edema confer neuroprotective effects and can improve neurobehavioral and cognitive outcomes in preclinical models of ICH (Rolland et al., 2011). Along these lines, Resveratrol treatment significantly attenuated acute cerebral edema and improved neurobehavioral outcomes after ICH.

The mechanism by which Resveratrol confers neuroprotection after ICH could be by directly activating SIRT1, as Resveratrol is regarded as a potent SIRT1 activator (Sinclair and Guarente, 2014). The sirtuin gene was first discovered in yeast as a transcription repressor and its mammalian orthologues, the sirtuins, are a family comprising seven members: SIRT1–SIRT7 (Frye, 2000). Sirtuins are categorized as class III histone deacetylases (Schemies et al., 2010). Apart from deacetylating histones, non-histone proteins are also substrates of sirtuins and they differ from other histone deacetylases in that, their activity is NAD+ dependent (Schemies et al., 2010). We recently reported the altered acetylation status of histone and non- histone proteins in the pathophysiology of ICH (Sukumari-Ramesh et al., 2016). Further, augmented activation of sirtuin 1 in neurons and microglia are known to attenuate oxidative neuronal injury and inflammatory response, respectively (Cho et al., 2015; Zhang et al., 2016). Notably, sirtuin 1 deficiency in microglia contributes to neurodegeneration via the epigenetic regulation of IL-1β (Cho et al., 2015). Consistently, Resveratrol treatment attenuated the brain levels of proinflammatory cytokines such as IL-1 β and TNF-α and reduced hypoxia-induced brain damage (Liu et al., 2007; Zhang et al., 2015; Jeong et al., 2016). Besides its anti-inflammatory properties, Resveratrol is a potent antioxidant and was found to be neuroprotective in both *in vitro* and *in vivo* models of hypoxia, excitotoxicity and ischemia partly due to its ability to scavenge free radicals and augment antioxidant enzymes (Virgili and Contestabile, 2000; Jang and Surh, 2003; Zhuang et al., 2003; Kiziltepe et al., 2004; Wang et al., 2004; Raval et al., 2006). The treatment with resveratrol immediately after traumatic brain injury attenuated oxidative brain damage and lesion volume in adult rats (Ates et al., 2007). Further, Resveratrol is also known to modulate Nrf2–Heme oxygenase 1 antioxidant signaling (Chen et al., 2005) and we recently reported the efficacy of post-injury administration of TBHQ, an inducer of Nrf2 in improving acute neurological outcomes after ICH (Sukumari-Ramesh and Alleyne, 2016). Furthermore, several studies have suggested that activation of SIRT1 may rescue mitochondrial function and inhibit apoptosis in cerebral ischemia and neurodegenerative diseases (Hernandez-Jimenez et al., 2013). Consistently, Resveratrol treatment attenuated the number of Fluoro Jade B and TUNEL positive cells after ICH. However, further studies required characterizing the precise molecular mechanism by which Resveratrol exerts neuroprotection after ICH.

Though Resveratrol is not known to be toxic or cause significant adverse effects in humans, the clinical use of Resveratrol is partly limited by its poor bioavailability (Walle, 2011; Francioso et al., 2014). One of the most efficacious ways to maximize absorption of resveratrol is to bypass the gastrointestinal tract and deliver directly into the bloodstream. Along these lines, the acute neuroprotective effects conferred by Resveratrol due to its intravenous administration after ICH, as demonstrated herein, has high translational relevance warranting future studies.

## Author contributions

FB carried out the immunohistochemical studies and participated in the data analysis. CA participated in project discussions. SS conceived and designed the experiments. SS also conducted the animal surgeries, data analysis and drafted the manuscript. All authors read and approved the final manuscript.

### Conflict of interest statement

The authors declare that the research was conducted in the absence of any commercial or financial relationships that could be construed as a potential conflict of interest.

## Supplementary material

Supplementary Data-Table 1 Resveratrol or vehicle was administered 30 min post-ICH and neurobehavioral outcome was estimated at 24 and 72 h post-ICH/sham by an independent researcher blinded to the experimental groups using a composite neurological test comprised of six neurobehavioral sub-tests (climbing, circling, compulsory circling, whisker response, bilateral grasp, and beam walking) and each sub-test was scored from 0 (performs with no impairment) to 4 (severe impairment) and the mean test scores are given. The data in Figures 1, 7 are derived from a composite neurological deficit score that was calculated as the sum of the scores on all the six sub-tests. *n* = 9–13/group.

## References

[B1] Aronowski J. Hall C. E. (2005). New horizons for primary intracerebral hemorrhage treatment: experience from preclinical studies. Neurol. Res. 27, 268–279. 10.1179/016164105X25225 15845210

[B2] Aronowski J. Zhao X. (2011). Molecular pathophysiology of cerebral hemorrhage: secondary brain injury. Stroke 42, 1781–1786. 10.1161/STROKEAHA.110.596718 21527759 PMC3123894

[B3] Ates O. Cayli S. Altinoz E. Gurses I. Yucel N. Kocak A.. (2006). Effects of resveratrol and methylprednisolone on biochemical, neurobehavioral and histopathological recovery after experimental spinal cord injury. Acta Pharmacol. Sin. 27, 1317–1325. 10.1111/j.1745-7254.2006.00416.x 17007738

[B4] Ates O. Cayli S. Altinoz E. Gurses I. Yucel N. Sener M.. (2007). Neuroprotection by resveratrol against traumatic brain injury in rats. Mol. Cell. Biochem. 294, 137–144. 10.1007/s11010-006-9253-0 16924419

[B5] Baur J. A. Sinclair D. A. (2006). Therapeutic potential of resveratrol: the *in vivo* evidence. Nat. Reviews Drug Disc. 5, 493–506. 10.1038/nrd2060 16732220

[B6] Bonsack F. Alleyne C. H. Sukumari-Ramesh S. (2016). Augmented expression of TSPO after intracerebral hemorrhage: a role in inflammation? J. Neuroinflammation 13:151. 10.1186/s12974-016-0619-2 27315802 PMC4912814

[B7] Broderick J. P. Brott T. G. Duldner J. E. Tomsick T. Huster G. (1993). Volume of intracerebral hemorrhage. A powerful and easy-to-use predictor of 30-day mortality. Stroke 24, 987–993. 10.1161/01.STR.24.7.987 8322400

[B8] Butcher K. S. Baird T. MacGregor L. Desmond P. Tress B. Davis S. (2004). Perihematomal edema in primary intracerebral hemorrhage is plasma derived. Stroke 35, 1879–1885. 10.1161/01.STR.0000131807.54742.1a 15178826

[B9] Chen C. Y. Jang J. H. Li M. H. Surh Y. J. (2005). Resveratrol upregulates heme oxygenase-1 expression via activation of NF-E2-related factor 2 in PC12 cells. Biochem. Biophys. Res. Commun. 331, 993–1000. 10.1016/j.bbrc.2005.03.237 15882976

[B10] Cho S. H. Chen J. A. Sayed F. Ward M. E. Gao F. Nguyen T. A.. (2015). SIRT1 deficiency in microglia contributes to cognitive decline in aging and neurodegeneration via epigenetic regulation of IL-1beta. J. Neurosci. 35, 807–818. 10.1523/JNEUROSCI.2939-14.2015 25589773 PMC4293425

[B11] Clark W. Gunion-Rinker L. Lessov N. Hazel K. (1998). Citicoline treatment for experimental intracerebral hemorrhage in mice. Stroke 29, 2136–2140. 10.1161/01.STR.29.10.2136 9756595

[B12] Flaherty M. L. Haverbusch M. Sekar P. Kissela B. Kleindorfer D. Moomaw C. J.. (2006). Long-term mortality after intracerebral hemorrhage. Neurology 66, 1182–1186. 10.1212/01.wnl.0000208400.08722.7c 16636234

[B13] Flemming K. D. Wijdicks E. F. Li H. (2001). Can we predict poor outcome at presentation in patients with lobar hemorrhage? Cerebrovasc. Dis. 11, 183–189. 10.1159/000047636 11306765

[B14] Flemming K. D. Wijdicks E. F. St Louis E. K. Li H. (1999). Predicting deterioration in patients with lobar haemorrhages. J. Neurol. Neurosurg. Psychiatr. 66, 600–605. 10.1136/jnnp.66.5.600 10209170 PMC1736365

[B15] Francioso A. Mastromarino P. Masci A. d'Erme M. Mosca L. (2014). Chemistry, stability and bioavailability of resveratrol. Med. Chem. 10, 237–245. 10.2174/15734064113096660053 24329932

[B16] Frye R. A. (2000). Phylogenetic classification of prokaryotic and eukaryotic Sir2-like proteins. Biochem. Biophys. Res. Commun. 273, 793–798. 10.1006/bbrc.2000.3000 10873683

[B17] Gebel J. M. Jr. Jauch E. C. Brott T. G. Khoury J. Sauerbeck L. Salisbury S.. (2002). Relative edema volume is a predictor of outcome in patients with hyperacute spontaneous intracerebral hemorrhage. Stroke 33, 2636–2641. 10.1161/01.STR.0000035283.34109.EA 12411654

[B18] Gupta Y. K. Briyal S. Chaudhary G. (2002). Protective effect of trans-resveratrol against kainic acid-induced seizures and oxidative stress in rats. Pharmacol. Biochem. Behav. 71, 245–249. 10.1016/S0091-3057(01)00663-3 11812529

[B19] Gupta Y. K. Chaudhary G. Sinha K. Srivastava A. K. (2001). Protective effect of resveratrol against intracortical FeCl3-induced model of posttraumatic seizures in rats. Methods Find. Exp. Clin. Pharmacol. 23, 241–244. 10.1358/mf.2001.23.5.662120 11712643

[B20] Gwak H. Kim S. Dhanasekaran D. N. Song Y. S. (2016). Resveratrol triggers ER stress-mediated apoptosis by disrupting N-linked glycosylation of proteins in ovarian cancer cells. Cancer Lett. 371, 347–353. 10.1016/j.canlet.2015.11.032 26704305

[B21] Hanley D. F. (2009). Intraventricular hemorrhage: severity factor and treatment target in spontaneous intracerebral hemorrhage. Stroke 40, 1533–1538. 10.1161/STROKEAHA.108.535419 19246695 PMC2744212

[B22] Hernandez-Jimenez M. Hurtado O. Cuartero M. I. Ballesteros I. Moraga A. Pradillo J. M.. (2013). Silent information regulator 1 protects the brain against cerebral ischemic damage. Stroke 44, 2333–2337. 10.1161/STROKEAHA.113.001715 23723308

[B23] Hoda U. Agarwal N. B. Vohora D. Parvez S. Raisuddin S. (2016). Resveratrol suppressed seizures by attenuating IL-1β, IL1-Ra, IL-6, and TNF-α in the hippocampus and cortex of kindled mice. Nutr. Neurosci. [Epub ahead of print]. 10.1080/1028415X.2016.1189057 27256583

[B24] Hu X. Tao C. Gan Q. Zheng J. Li H. You C. (2016). Oxidative stress in intracerebral hemorrhage: sources, mechanisms, and therapeutic targets. Oxid. Med. Cell. Longev., 2016:3215391. 10.1155/2016/3215391 26843907 PMC4710930

[B25] Jang J. H. Surh Y. J. (2003). Protective effect of resveratrol on beta-amyloid-induced oxidative PC12 cell death. Free Radic. Biol. Med. 34, 1100–1110. 10.1016/S0891-5849(03)00062-5 12684095

[B26] Jeong S. I. Shin J. A. Cho S. Kim H. W. Lee J. Y. Kang J. L.. (2016). Resveratrol attenuates peripheral and brain inflammation and reduces ischemic brain injury in aged female mice. Neurobiol. Aging 44, 74–84. 10.1016/j.neurobiolaging.2016.04.007 27318135

[B27] Kaplan S. Bisleri G. Morgan J. A. Cheema F. H. Oz M. C. (2005). Resveratrol, a natural red wine polyphenol, reduces ischemia-reperfusion-induced spinal cord injury. Ann. Thorac. Surg. 80, 2242–2249. 10.1016/j.athoracsur.2005.05.016 16305881

[B28] Ke K. Song Y. Shen J. Niu M. Zhang H. Yuan D.. (2015). Up-regulation of Glis2 involves in neuronal apoptosis after intracerebral hemorrhage in adult rats. Cell. Mol. Neurobiol. 35, 345–354. 10.1007/s10571-014-0130-1 25370802 PMC11486247

[B29] Keep R. F. Hua Y. Xi G. (2012). Intracerebral haemorrhage: mechanisms of injury and therapeutic targets. Lancet Neurol. 11, 720–731. 10.1016/S1474-4422(12)70104-7 22698888 PMC3884550

[B30] King M. D. McCracken D. J. Wade F. M. Meiler S. E. Alleyne C. H. Jr. Dhandapani K. M. (2011). Attenuation of hematoma size and neurological injury with curcumin following intracerebral hemorrhage in mice. J. Neurosurg. 115, 116–123. 10.3171/2011.2.JNS10784 21417704 PMC3153730

[B31] Kiziltepe U. Turan N. N. Han U. Ulus A. T. Akar F. (2004). Resveratrol, a red wine polyphenol, protects spinal cord from ischemia-reperfusion injury. J. Vasc. Surg. 40, 138–145. 10.1016/j.jvs.2004.03.032 15218474

[B32] Kothari R. U. Brott T. Broderick J. P. Barsan W. G. Sauerbeck L. R. Zuccarello M.. (1996). The ABCs of measuring intracerebral hemorrhage volumes. Stroke 27, 1304–1305. 10.1161/01.STR.27.8.1304 8711791

[B33] Leclerc J. L. Lampert A. S. Diller M. A. Immergluck J. B. Dore S. (2015). Prostaglandin E2 EP2 receptor deletion attenuates intracerebral hemorrhage-induced brain injury and improves functional recovery. ASN Neuro 7:1759091415578713. 10.1177/1759091415578713 25873308 PMC4720177

[B34] Li X. M. Zhou M. T. Wang X. M. Ji M. H. Zhou Z. Q. Yang J. J. (2014). Resveratrol pretreatment attenuates the isoflurane-induced cognitive impairment through its anti-inflammation and -apoptosis actions in aged mice. J. Mol. Neurosci. 52, 286–293. 10.1007/s12031-013-0141-2 24126892

[B35] Liu Y. G. Wang X. D. Zhang X. B. (2007). Effects of resveratrol on inflammatory process induced by focal cerebral ischemia-reperfusion in rats. Zhongguo Zhong Yao Za Zhi, 32, 1792–1795. 17993005

[B36] Lok J. Zhao S. Leung W. Seo J. H. Navaratna D. Wang X.. (2012). Neuregulin-1 effects on endothelial and blood-brain-barrier permeability after experimental injury. Transl. Stroke Res. 3(Suppl. 1), S119–S124. 10.1007/s12975-012-0157-x 22773936 PMC3389802

[B37] Lopez M. S. Dempsey R. J. Vemuganti R. (2015). Resveratrol neuroprotection in stroke and traumatic CNS injury. Neurochem. Int. 89, 75–82. 10.1016/j.neuint.2015.08.009 26277384 PMC4587342

[B38] Mark L. Nikfardjam M. S. Avar P. Ohmacht R. (2005). A validated HPLC method for the quantitative analysis of trans-resveratrol and trans-piceid in Hungarian wines. J. Chromatogr. Sci. 43, 445–449. 10.1093/chromsci/43.9.445 16212788

[B39] Masada T. Hua Y. Xi G. Yang G. Y. Hoff J. T. Keep R. F. (2001). Attenuation of intracerebral hemorrhage and thrombin-induced brain edema by overexpression of interleukin-1 receptor antagonist. J. Neurosurg. 95, 680–686. 10.3171/jns.2001.95.4.0680 11596963

[B40] Mukherjee S. Dudley J. I. Das D. K. (2010). Dose-dependency of resveratrol in providing health benefits. Dose Response 8, 478–500. 10.2203/dose-response.09-015.Mukherjee 21191486 PMC2990065

[B41] Narayanan S. V. Dave K. R. Saul I. Perez-Pinzon M. A. (2015). Resveratrol preconditioning protects against cerebral ischemic injury via nuclear Erythroid 2-Related Factor 2. Stroke 46, 1626–1632. 10.1161/STROKEAHA.115.008921 25908459 PMC4442036

[B42] Parker J. A. Arango M. Abderrahmane S. Lambert E. Tourette C. Catoire H.. (2005). Resveratrol rescues mutant polyglutamine cytotoxicity in nematode and mammalian neurons. Nat. Genet. 37, 349–350. 10.1038/ng1534 15793589

[B43] Qureshi A. I. Tuhrim S. Broderick J. P. Batjer H. H. Hondo H. Hanley D. F. (2001). Spontaneous intracerebral hemorrhage. N. Engl. J. Med. 344, 1450–1460. 10.1056/NEJM200105103441907 11346811

[B44] Raval A. P. Dave K. R. Perez-Pinzon M. A. (2006). Resveratrol mimics ischemic preconditioning in the brain. J. Cereb. Blood Flow Metab. 26, 1141–1147. 10.1038/sj.jcbfm.9600262 16395277

[B45] Rolland W. B. II. Manaenko A. Lekic T. Hasegawa Y. Ostrowski R. Tang J.. (2011). FTY720 is neuroprotective and improves functional outcomes after intracerebral hemorrhage in mice. Acta Neurochir. Suppl. 111, 213–217. 10.1007/978-3-7091-0693-8_36 21725758 PMC3569072

[B46] Rosenberg G. A. Mun-Bryce S. Wesley M. Kornfeld M. (1990). Collagenase-induced intracerebral hemorrhage in rats. Stroke 21, 801–807. 10.1161/01.STR.21.5.801 2160142

[B47] Schemies J. Uciechowska U. Sippl W. Jung M. (2010). NAD(+) -dependent histone deacetylases (sirtuins) as novel therapeutic targets. Med. Res. Rev. 30, 861–889. 10.1002/med.20178 19824050

[B48] Shao A. W. Wu H. J. Chen S. Ammar A. B. Zhang J. M. Hong Y. (2014). Resveratrol attenuates early brain injury after subarachnoid hemorrhage through inhibition of NF-kappaB-dependent inflammatory/MMP-9 pathway. CNS Neurosci. Ther. 20, 182–185. 10.1111/cns.12194 24279692 PMC6493158

[B49] Sinclair D. A. Guarente L. (2014). Small-molecule allosteric activators of sirtuins. Annu. Rev. Pharmacol. Toxicol. 54, 363–380. 10.1146/annurev-pharmtox-010611-134657 24160699 PMC4018738

[B50] Sukumari-Ramesh S. Alleyne C. H. Jr. (2016). Post-injury administration of tert-butylhydroquinone attenuates acute neurological injury after intracerebral hemorrhage in mice. J. Mol. Neurosci. 58, 525–531. 10.1007/s12031-016-0722-y 26867538

[B51] Sukumari-Ramesh S. Alleyne C. H. Jr. Dhandapani K. M. (2012a). Astrocyte-specific expression of survivin after intracerebral hemorrhage in mice: a possible role in reactive gliosis? J. Neurotrauma, 29, 2798–2804. 10.1089/neu.2011.2243 22862734 PMC3521135

[B52] Sukumari-Ramesh S. Alleyne C. H. Jr. Dhandapani K. M. (2012b). Astrogliosis: a target for intervention in intracerebral hemorrhage? Transl. Stroke Res. 3(Suppl. 1), 80–87. 10.1007/s12975-012-0165-x 24323864

[B53] Sukumari-Ramesh S. Alleyne C. H. Jr. Dhandapani K. M. (2016). The histone deacetylase inhibitor suberoylanilide hydroxamic acid (SAHA) confers acute neuroprotection after intracerebral hemorrhage in mice. Transl. Stroke Res. 7, 141–148. 10.1007/s12975-015-0421-y 26338677

[B54] Taguchi K. Hida M. Matsumoto T. Kobayashi T. (2015). Resveratrol Ameliorates Clonidine-Induced Endothelium-Dependent Relaxation Involving Akt and Endothelial Nitric Oxide Synthase Regulation in Type 2 Diabetic Mice. Biol. Pharm. Bull. 38, 1864–1872. 10.1248/bpb.b15-00403 26632178

[B55] Tellone E. Galtieri A. Russo A. Giardina B. Ficarra S. (2015). Resveratrol: a focus on several neurodegenerative diseases. Oxid. Med. Cell. Longev. 2015:392169. 10.1155/2015/392169 26180587 PMC4477222

[B56] Tsai T. Y. Chen T. C. Wang I. J. Yeh C. Y. Su M. J. Chen R. H.. (2015). The effect of resveratrol on protecting corneal epithelial cells from cytotoxicity caused by moxifloxacin and benzalkonium chloride. Invest. Ophthalmol. Vis. Sci. 56, 1575–1584. 10.1167/iovs.14-15708 25670486

[B57] Urday S. Beslow L. A. Goldstein D. W. Vashkevich A. Ayres A. M. Battey T. W.. (2015a). Measurement of perihematomal edema in intracerebral hemorrhage. Stroke 46, 1116–1119. 10.1161/STROKEAHA.114.007565 25721012 PMC5340416

[B58] Urday S. Kimberly W. T. Beslow L. A. Vortmeyer A. O. Selim M. H. Rosand J.. (2015b). Targeting secondary injury in intracerebral haemorrhage–perihaematomal oedema. Nat. Rev. Neurol. 11, 111–122. 10.1038/nrneurol.2014.264 25623787

[B59] Virgili M. Contestabile A. (2000). Partial neuroprotection of *in vivo* excitotoxic brain damage by chronic administration of the red wine antioxidant agent, trans-resveratrol in rats. Neurosci. Lett. 281, 123–126. 10.1016/S0304-3940(00)00820-X 10704758

[B60] Wagner K. R. Sharp F. R. Ardizzone T. D. Lu A. Clark J. F. (2003). Heme and iron metabolism: role in cerebral hemorrhage. J. Cereb. Blood Flow Metab. 23, 629–652. 10.1097/01.WCB.0000073905.87928.6D 12796711

[B61] Walle T. (2011). Bioavailability of resveratrol. Ann. N.Y. Acad. Sci. 1215, 9–15. 10.1111/j.1749-6632.2010.05842.x 21261636

[B62] Wang C. W. Liu Y. J. Lee Y. H. Hueng D. Y. Fan H. C. Yang F. C.. (2014). Hematoma shape, hematoma size, Glasgow coma scale score and ICH score: which predicts the 30-day mortality better for intracerebral hematoma? PLoS ONE 9:e102326. 10.1371/journal.pone.0102326 25029592 PMC4100880

[B63] Wang J. Tsirka S. E. (2005). Neuroprotection by inhibition of matrix metalloproteinases in a mouse model of intracerebral haemorrhage. Brain 128(Pt 7), 1622–1633. 10.1093/brain/awh489 15800021

[B64] Wang Q. Xu J. Rottinghaus G. E. Simonyi A. Lubahn D. Sun G. Y.. (2002). Resveratrol protects against global cerebral ischemic injury in gerbils. Brain Res. 958, 439–447. 10.1016/S0006-8993(02)03543-6 12470882

[B65] Wang Q. Yu S. Simonyi A. Rottinghaus G. Sun G. Y. Sun A. Y. (2004). Resveratrol protects against neurotoxicity induced by kainic acid. Neurochem. Res. 29, 2105–2112. 10.1007/s11064-004-6883-z 15662844

[B66] Wasserman J. K. Schlichter L. C. (2007). Minocycline protects the blood-brain barrier and reduces edema following intracerebral hemorrhage in the rat. Exp. Neurol. 207, 227–237. 10.1016/j.expneurol.2007.06.025 17698063

[B67] Wei C. C. Kong Y. Y. Li G. Q. Guan Y. F. Wang P. Miao C. Y. (2017). Nicotinamide mononucleotide attenuates brain injury after intracerebral hemorrhage by activating Nrf2/HO-1 signaling pathway. Sci. Rep. 7:717. 10.1038/s41598-017-00851-z 28386082 PMC5429727

[B68] West T. Atzeva M. Holtzman D. M. (2007). Pomegranate polyphenols and resveratrol protect the neonatal brain against hypoxic-ischemic injury. Dev. Neurosci. 29, 363–372. 10.1159/000105477 17762204 PMC3066259

[B69] Wu Z. Xu Q. Zhang L. Kong D. Ma R. Wang L. (2009). Protective effect of resveratrol against kainate-induced temporal lobe epilepsy in rats. Neurochem. Res. 34, 1393–1400. 10.1007/s11064-009-9920-0 19219549

[B70] Xi G. Keep R. F. Hoff J. T. (2006). Mechanisms of brain injury after intracerebral haemorrhage. Lancet Neurol. 5, 53–63. 10.1016/S1474-4422(05)70283-0 16361023

[B71] Yang Y. B. Piao Y. J. (2003). Effects of resveratrol on secondary damages after acute spinal cord injury in rats. Acta Pharmacol. Sin. 24, 703–710. Available online at: http://www.chinaphar.com/article/view/9182/9846 12852839

[B72] Zhang Q. Yuan L. Zhang Q. Gao Y. Liu G. Xiu M.. (2015). Resveratrol attenuates hypoxia-induced neurotoxicity through inhibiting microglial activation. Int. Immunopharmacol. 28, 578–587. 10.1016/j.intimp.2015.07.027 26225925

[B73] Zhang X. S. Wu Q. Wu L. Y. Ye Z. N. Jiang T. W. Li W.. (2016). Sirtuin 1 activation protects against early brain injury after experimental subarachnoid hemorrhage in rats. Cell Death Dis. 7, e2416. 10.1038/cddis.2016.292 27735947 PMC5133967

[B74] Zheng H. Chen C. Zhang J. Hu Z. (2016). Mechanism and Therapy of Brain Edema after Intracerebral Hemorrhage. Cerebrovasc. Dis. 42, 155–169. 10.1159/000445170 27110940

[B75] Zhuang H. Kim Y.-S. Koehler R. C. Doré S. (2003). Potential mechanism by which resveratrol, a red wine constituent, protects neurons. Ann. N.Y. Acad. Sci. 993, 276–286; discussion 287–278. 10.1111/j.1749-6632.2003.tb07534.x 12853318

