# Supplementary material for: Resveratrol Attenuates Neurodegeneration and Improves Neurological Outcomes after Intracerebral Hemorrhage in Mice
Source: Front Cell Neurosci. 2017 Aug 8;11:228. doi: 10.3389/fncel.2017.00228 (PMC5550718; doi:10.3389/fncel.2017.00228)
Supplement: Supplementary Data-Table 1 — Resveratrol or vehicle was administered 30 min post-ICH and neurobehavioral outcome was estimated at 24 and 72 h post-ICH/sham by an independent researcher blinded to the experimental groups using a composite neurological test comprised of six neurobehavioral sub-tests (climbing, circling, compulsory circling, whisker response, bilateral grasp, and beam walking) and each sub-test was scored from 0 (performs with no impairment) to 4 (severe impairment) and the mean test scores are given. The data in Figures 1, 7 are derived from a composite neurological deficit score that was calculated as the sum of the scores on all the six sub-tests. n = 9–13/group. [file Table1.pdf]

**Supplementary Data- Table 1**

| | 24 h post-ICH/Sham | | | 72 h post-ICH/Sham | | |
|---|---|---|---|---|---|---|
| **Sub-test** | **Sham** | **ICH** | **ICH + Resveratrol** | **Sham** | **ICH** | **ICH + Resveratrol** |
| Circling | 0 | 0.56 | 0.31 | 0 | 0 | 0 |
| Compulsory Circling | 0 | 0.78 | 0.69 | 0 | 1.7 | 0.56 |
| Beam Walking | 0.56 | 3.2 | 2.4 | 0.11 | 1.7 | 1.9 |
| Whisker Response | 0.56 | 3.1 | 1.8 | 0.11 | 1.6 | 0.44 |
| Climbing | 0.11 | 2.2 | 0.31 | 0 | 0.11 | 0.11 |
| Bilateral Grasp | 0.22 | 1.4 | 1 | 0.11 | 3.1 | 1.1 |
